# COVID-19 Vaccine Hesitancy among the General Population: A Cross-Sectional Study

**DOI:** 10.3390/vaccines11061125

**Published:** 2023-06-20

**Authors:** Sawsan Mustafa Abdalla, Elsadig Yousif Mohamed, Hala Mostafa Elsabagh, Mohammad Shakil Ahmad, Riyaz Ahamed Shaik, Vini Mehta, Ankita Mathur, Sharad Balasaheb Ghatge

**Affiliations:** 1 Department of Family and Community Medicine, College of Medicine, Majmaah University, Al-Majmaah 11952, Saudi Arabia; 2 Basic Medical Sciences Department, College of Dentistry, Majmaah University, Al-Majmaah 11952, Saudi Arabia; 3 Department of Public Health and Community Medicine, Tanta University, Tanta 6632110, Egypt; 4 Department of Public Health Dentistry, Dr. D.Y. Patil Dental College and Hospital, Dr. D.Y. Patil Vidyapeeth, Pune 411018, Maharashtra, India; 5 Department of Periodontology, Dr. D.Y. Patil Dental College and Hospital, Dr. D.Y. Patil Vidyapeeth, Pune 411018, Maharashtra, India; amathur@statsense.in; 6 Division of Interventional Radiology, Department of Radiology and Imaging, Grant Government Medical College, Sir JJ Group of Hospitals, Mumbai 400008, Maharashtra, India; 7 Department of Interventional Neuroradiology, Bombay Hospital, Mumbai 400020, Maharashtra, India

**Keywords:** vaccine, hesitancy, fear, mistrust

## Abstract

Hesitancy about receiving vaccines has been deemed a global danger to public health by WHO. The sociocultural backgrounds of the people have an impact on vaccine acceptance. The purpose of this study was to examine the effect of sociodemographic factors on COVID-19 vaccination hesitancy as well as to identify the factors that contributed to COVID-19 vaccine hesitancy. Methods: A cross-sectional study was carried out to determine the primary variables causing COVID-19 vaccination hesitancy among residents of Pune. The general population was sampled through simple random sampling. The minimum sample size was determined to be 1246. The questionnaire inquired about the individuals’ sociodemographic information, vaccination status, and reasons for vaccine hesitancy. Results: In total, there were 5381 subjects, 1669 of whom were unvaccinated and 3712 of whom were partially vaccinated. Fear of adverse effects (51.71%), fear of losing a few days of work (43.02%), and inability to secure a vaccine slot online (33.01%) were the most frequently cited reasons. An older population (>60 years, *p* = 0.004), males (*p* = 0.032), those who were literate (*p* = 0.011), those of lower middle socioeconomic status (*p* = 0.001), and smokers were significantly associated with fear and mistrust of the COVID-19 vaccine, while mistrust of the vaccine was greatest among individuals from the upper and lower middle classes (*p* = 0.001). Conclusion: Vaccine hesitancy due to concerns about the side effects and long-term complications was prevalent among the elderly, males, those from the lower middle class, and smokers. This study emphasizes the importance of communicating effectively about the vaccine’s efficacy, its distribution, and vaccination sites.

## 1. Introduction

As of 3 February 2023, there have been more than 754 million reported infections with SARS-CoV-2 and nearly 6.8 million reported deaths from COVID-19 [1]. To curb the infection, quarantines and lockdowns were imposed, but these measures proved ineffective in containing the infection and resulted in a global economic slowdown. Additionally, the immunity threshold required for herd immunity is currently unknown, but several studies have suggested that it is between 71% and 74%, though the recent appearance of more infectious variants may raise this figure [2,3,4]. In May 2020, the 73rd World Health Assembly issued a resolution recognizing the role of extensive immunization as a global goal of public health for preventing, containing, and stopping the transmission of SARS-CoV-2 [3].

People’s reluctance to receive safe and recommended available vaccines, known as ‘vaccine hesitancy’, was already a growing concern before the COVID-19 pandemic. Several studies have been carried out to find out about the acceptance rate of the COVID-19 vaccine among the general population [5,6,7,8]. The acceptance of vaccines depends on the sociocultural background of the individuals. Moreover, their perspectives are influenced by their peers, self-help groups, and the information available to them through various sources, and their reliability and authenticity. Apart from the factors related to individuals and the community, administrative issues also influence vaccine uptake. These issues are related to the vaccine’s production and distribution, information about the free availability of the vaccine, its size, and the nature of the vaccine, etc. Systematic review studies with emphasis on the COVID-19 vaccine’s perceptions and levels of acceptability in developing countries have been carried out which can help in decision-making and boost the trust in medical authorities [9,10].

Strategies to combat vaccination rejection must be developed after careful consideration of a regional assessment of people’s preference for the COVID-19 vaccine [11]. However, research on the community preferences, willingness, and concerns regarding vaccination against COVID-19 is lacking in India. With this background, this study aimed to identify the various factors leading to COVID-19 vaccine hesitancy assessed at four different levels: fear, mistrust, administrative issues, and awareness among the general population. Secondly, it also attempted to evaluate the impact of sociodemographic factors on COVID-19 vaccine hesitancy and determine the influence of the factors of second-dose COVID-19 vaccine hesitancy.

## 2. Materials and Methods

A cross-sectional study was conducted amongst the general population of Pune city, who were aged above 18 years. Consenting adults willing to be part of the study were included as study participants. The sample size was calculated on the basis of a previous study conducted on real-time data obtained from Indian states on the Cowin dashboard and the Ministry of Health and Family Welfare, India. This study estimated that 29% of individuals showed vaccine hesitancy [12]. The minimum sample size was calculated to be 1246 at a 99.9% confidence interval.

The data were collected through face-to-face interviews. The general population was sampled through simple random sampling. There are 15 blocks in total in Pune city. Approximately 350 individuals were randomly interviewed from each block. The questionnaire was designed and pretested by the researchers after an extensive literature review. In the first part of the questionnaire, the participants were informed about the purpose of the research, and their consent was obtained. After the study, participants agreed to participate and gave consent, and the questionnaire was administered to them. The questionnaire consisted of questions about the sociodemographic characteristics of the participants and their thoughts about possible COVID-19 vaccines. In the first part of the questionnaire, questions regarding demographic variables such as age, gender, educational status, occupation, residence, economic status, the presence of children, lifestyle factors such as smoking, alcohol intake, diet, and chronic illness/comorbidities were included. The modified Kuppuswamy socioeconomic scale (MKS) was used to categorize families into five classes in the urban area: upper class, upper middle class, lower middle class, upper lower class, and lower class. This scale is scored on the basis of the monthly income of the family, and the education and occupation of the head of the family. The scale is updated each year to adjust for monetary inflation [13].

The second part of the questionnaire contains around 20 propositions regarding the COVID-19 vaccine divided into four different factors: fear, mistrust, awareness, and administrative and other factors. For each statement, yes/no options were included. The third part of the questionnaire consists of reasons for hesitancy about the second-dose COVID-19 vaccine. For each statement, yes/no options were included. The questionnaire also included questions about previous infection with COVID-19, vaccination against seasonal influenza, and knowledge and information sources about COVID-19. Lastly, the level of acceptance or hesitancy about the COVID-19 vaccine, beliefs regarding COVID-19 vaccination, and the type of COVID-19 vaccine preferred.

The data were inputted into Microsoft Excel, and all the analyses were carried out using R software (version 4.1.2). The sociodemographic features of the individuals were expressed as the frequency and proportion for categorical data, and the means and standard deviations were used for continuous data. The associations of vaccination status, fear, and mistrust with sociodemographic characteristics were analyzed using the chi-square test.

In order to assess the predictors of vaccine hesitancy, the responses to the 20 questions in the questionnaire were scored as 0 and 1 and summed up. The total score of each individual was categorized as mild (≤8), moderate (9–15), and high vaccine hesitancy (score ≥ 16). To perform the multivariate logistic regression analysis, the ordered categories were reclassified as less hesitant (scores ≤ 15) and high hesitant (scores ≥ 16), as there were fewer mild cases. *p*-values < 0.005 were considered to be statistically significant.

## 3. Results

Out of 5411 participants, 1669 (31.82%) were unvaccinated and 3682 (68.04%) were partially vaccinated. The following table gives the distribution of variables over the unvaccinated and partially vaccinated groups. The maximum number of participants in both groups belonged to the group aged 21 to 30 years (unvaccinated group: 817, 49%; partially vaccinated group: 1516, 40.8%). A preponderance of males over females was seen in both the unvaccinated (1123, 67.3%) and partially vaccinated groups (2181, 58.8%). In comparison with 30.1% of the participants in the unvaccinated groups being illiterate, only about one-fifth (22.3%) of the individuals were illiterate in the partially vaccinated group. More than half of the unvaccinated individuals (52.7%) belonged to the upper lower class, while 46.5% of partially vaccinated individuals were in the upper lower class. A history of previous COVID-19 infection was reported in 8.6% of the unvaccinated and 12.6% of the partially vaccinated individuals. The most common source of information among partially vaccinated individuals (45.7%) was healthcare workers, while that among unvaccinated individuals was electronic media (37.9%). Although the history of annual influenza vaccination was almost similar in both subgroups, a lack of awareness was more common among unvaccinated individuals (42.1%) compared with those who were partially vaccinated (35.9%) (Table 1).

Out of 1669 unvaccinated subjects, 863 (51.71%) feared that the vaccine would cause adverse events, 391 (23.43%) feared that the vaccine would cause COVID-19 infections, 313 (18.8%) feared that the vaccine might cause death, 718 (43.0%) feared that the vaccination would cause the loss of a few days of work, and 216 (12.9%) feared that the vaccine might lead to long-term side effects. Moreover 222 (13.3%) of the subjects did not trust the Indian COVID-19 vaccine, and 145 (8.69%) had suspicions of profiteering by the pharmaceutical industry. In addition, 340 (20.4%) lacked awareness of their eligibility for the vaccine, and 79 (4.7%) had previously been infected with COVID-19 and believed that the vaccine was not needed. Finally, 551 (33%) did not receive the vaccination due to the inability to book a slot for the vaccine online.

The barriers to vaccination in the unvaccinated group were in four categories: fear, mistrust, administrative issues, and awareness. The most common reason for non-acceptance of the vaccine was fear (90.7%). The fear of adverse effects was reported by about 51.7% of the individuals. About one-fourth of the individuals felt that the vaccine would cause a COVID-19 infection (23.4%) or even death (18.8%). About 43.0% feared the loss of work. Mistrust was reported by 61.2% of unvaccinated individuals. Doubts about vaccine efficacy were raised by 32.8% of the individuals. Around 14.6% felt that the vaccine was rolled out early without proper studies. More than 10% (13.3%) expressed their lack of trust in the Indian vaccine, and 8.7% of the individuals felt that pharmaceutical companies were making a profit out of the adverse situation.

**Figure 1:**
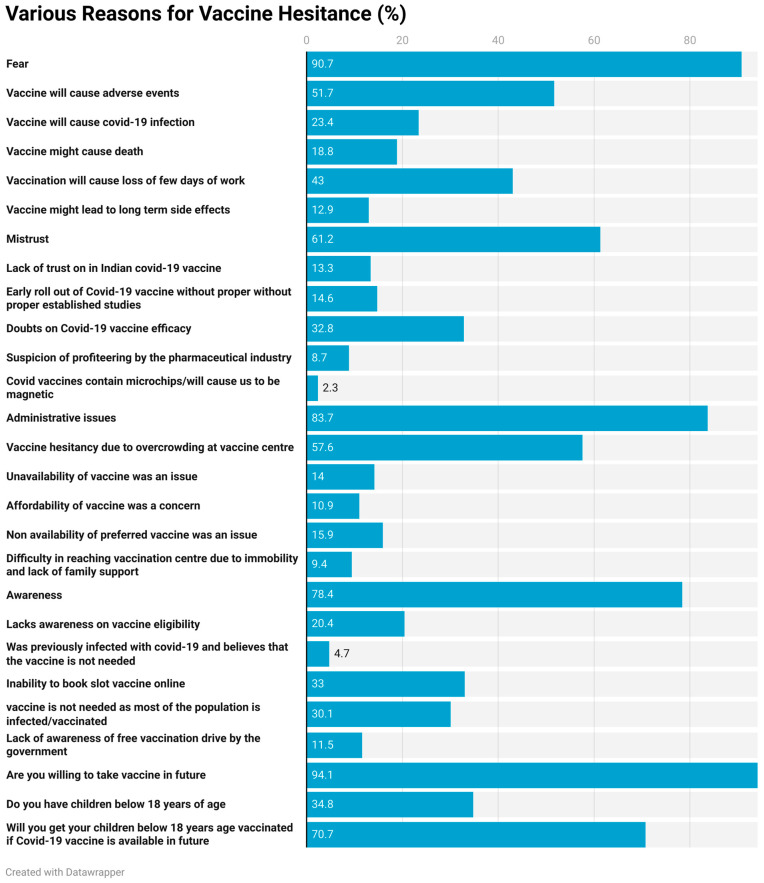
Various reasons for vaccine hesitancy.

The next most common reasons cited were related to administrative issues. The most common barriers were overcrowding (57.6%), unavailability of the vaccine (14%), and choice of vaccine (15.9%). Affordability was cited as a concern by 10.9%, and difficulty in reaching the vaccination site was mentioned by 9.4% of unvaccinated individuals. Overall, barriers of awareness were observed in 78.4% of individuals. These were a lack of awareness of vaccine eligibility (20.4%), inability to book a slot (33%), and lack of awareness regarding the free vaccination campaign (11.5%). About 30.1% felt that the vaccine was not needed because the individuals had acquired immunity through an infection or vaccination. Moreover, 94.1% of the individuals were willing to be vaccinated in the future and 70.7% of the individuals were ready to have their children vaccinated (Figure 1).

**Figure 2:**
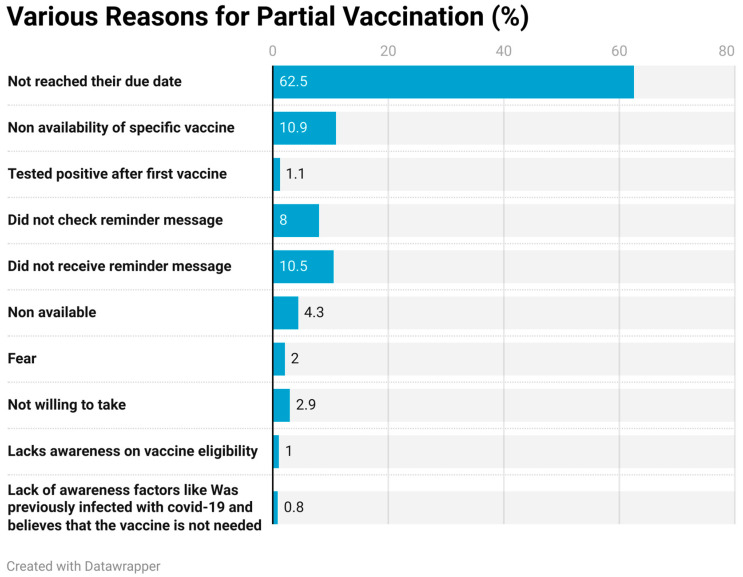
Various reasons for partial vaccination.

Out of 3712 partially vaccinated subjects, 2320 (62.5%) had not reached their due date, 42 (1.1%) tested positive after the first vaccine, 297 (8%) had not checked reminder messages, 389 (10.5%) did not receive a reminder message, 76 (2.0%) were afraid, 108 (2.9%) were not willing to take it, and 37 (1%) lacked awareness of their eligibility for the vaccine (Figure 2).

Fear and mistrust towards the COVID-19 vaccine were significantly associated with an older population (>60 years, *p* = 0.001; >60 years, *p* < 0.001). The male gender had more fear of vaccination than females (*p* = 0.032). Illiterate individuals (93.4%) were more fearful of vaccines than those who were literate (83.3%) (*p* = 0.011). Individuals belonging to lower levels of society were more fearful than other classes (*p* < 0.001), and mistrust towards the vaccine was highest among lower (96.6%) class individuals (*p* < 0.001). It was observed that the individuals with an addiction to smoking were more fearful (*p* = 0.045) and had greater mistrust of the vaccines than non-smokers (*p* < 0.001). Those individuals whose source of information was print media had higher levels of fear (20%) and half of those (50.1%) relying on electronic media had more mistrust than others (*p* < 0.001). A lack of awareness and administrative issues were significantly associated with the older population, male gender, and unemployed individuals. These factors of vaccine hesitancy were also significantly associated with upper middle populations and smokers (Table 2).

A logistic regression analysis was carried out to predict the factors associated with vaccine hesitancy. The three categories of scores for hesitancy, namely mild (≤8), moderate (9–15), and high vaccine hesitancy (≥16) were reclassified because there were fewer mild cases as follows: scores of ≤15, less hesitant; scores of ≥16, highly hesitant.

Both the univariate and multivariate models showed that age, literacy, income, socioeconomic status, and a history of smoking were the most significant factors for vaccine hesitancy. It was observed that vaccine hesitancy was higher in those aged 50 and above. The adjusted OR was 3.08 (95%: 1.28–7.39) and 6.52 (95%: 1.98–21.46) times higher in those aged 51–60 and above 60, respectively, compared to those aged <20 years.

Graduates and those with higher education were predominantly highly hesitant about the vaccine compared with illiterates (adj OR: 1.86; 95% CI: 1.08–3.22). Higher-income groups had higher hesitancy than lower-income groups. The adjusted OR of higher-income groups (7887–26,354 and >26,355) was significantly higher (OR: 16.76; 95%: 8.40–33.43; OR: 88.23, 95%: 35.83–217.28, respectively) than those from lower-income groups. Smokers had higher vaccine hesitancy than non-smokers (adj OR: 2.20, 95%: 1.59–3.04) (Table 3).

## 4. Discussion

The importance of vaccines in containing the infection was demonstrated mathematically through a robust fractional order model. The study ascertained that with the increasing efficacy of the vaccine, the number of infected individuals decreased [12]. In spite of this, vaccine hesitancy is one of several challenges in combating the novel coronavirus disease (COVID-19). Previous literature has ascertained that the problem of vaccine hesitancy is global, with wide variation in its prevalence ranging between 30% and 40% [14,15]. The present study was undertaken to explore the reasons for the non-acceptance of the COVID-19 vaccine among the unvaccinated population and partially vaccinated population. The willingness of unvaccinated individuals to be inoculated was 90%. Almost similar results were presented by a study conducted in seven provinces of Indonesia [16]. The study reported that 93.3% of the population was ready to be vaccinated. However, a study conducted on 370 Chileans found that only 49% of participants were open to vaccination [17]. This emphasizes that the population is supportive of vaccination, and the free availability of the vaccine has further strengthened their willingness to get vaccinated.

Most of the study’s participants were aged 21–40 years, with a preponderance of males from urban settings. Most of them were either illiterate or had completed education up to senior secondary level. The participants were either unemployed or semi-skilled workers. Most of them belonged to the upper lower or lower middle socioeconomic class. The growing body of evidence suggested that the likelihood of intention to be vaccinated was uniformly lower among women, younger people, those with lower incomes or educational levels, and members of ethnic minorities [15,18]. A nationwide study conducted in Qatar observed that women were both more resistant and hesitant to vaccination [19].

A similar trend across different studies reported in a systematic review by Ayyalasoma-yajula et al. found that females expressed greater vaccine hesitancy than males [20]. Women from a lower social class are dependent on their family members to make decisions about vaccination. Furthermore, they also had fears about being vaccinated. A study conducted in Nigeria ascertained that women had to obtain permission from their male partners, even for immunizing children [21]. This indirectly had an impact on the vaccination of their children as well. The vaccine hesitancy among lower social classes is caused by an interplay of various factors.

The barriers to acceptance of the COVID-19 vaccine were categorized as fear (90%), administrative issues (83.7%), awareness (78.4%), and mistrust (61%). The perceived fear was attributed to the adverse effects of the vaccine. One of the most common reasons for non-acceptance was a fear of adverse effects (51.7%). Almost one-fourth of the individuals thought they could contract COVID-19 after being vaccinated and 18.7% even feared death. Next, they had doubts about the efficacy of the vaccine (32.7%) and thought that proper studies had not been conducted to prove the efficacy of the vaccine (14.6%). Less than 10% had the view that this vaccine was just a stunt by the pharmaceutical companies. These results are consistent with other studies on migrants and low-income countries, which identified mistrust of the vaccines and concern over the side effects as the main causes [22,23]. Inadequate vaccine safety and fear about the side effects were the main causes of vaccine reluctance, according to a study conducted in Italy [24]. According to a Polish study, many avoid vaccinations out of concern about the potential side effects [25].

According to a qualitative study carried out in urban slums, many people living there were unaware of the benefits and necessity of vaccinations. The effectiveness of immunizations in containing the epidemic is still questioned by many. Some people think that receiving the vaccine might not be worth it [26]. Other major reasons for the non-acceptance of the vaccine were due to the loss of wages (43%) and the inability to book a slot for the vaccination (33%). Almost similar findings were reported in a study conducted in informal urban settlements of four metropolitan cities in India [27]. Most of the individuals in the lower social class are on a daily wage and they cannot afford to miss a single day at work in an already financially turbulent situation. Moreover, the lack of access to social help groups and a lack of information about free vaccination drives and how to book a slot were factors that interplayed and thereby affected the decision of vaccination [28,29].

The present study ascertained that the non-acceptance of the vaccine among the older population and individuals from the upper middle class was attributed to all four sub-categories: fear, mistrust, administrative issues, and awareness. More than half of the individuals were not compliant with the vaccine because of overcrowding at the vaccination sites. The older population, especially the retired personnel, had a lower perceived risk of infection. The assumption that the vaccination sites would be overcrowded further lowered their willingness to be vaccinated. Additionally, this population is particularly vulnerable to developing severe forms of COVID-19. As a result, they do not want to travel long distances, reducing their chances of becoming infected.

Moreover, hesitancy in vaccinating children was observed among 30% of the participants. A study reported that increased odds of vaccine hesitancy were specifically observed among parents with higher education [28]. Evidence suggests that parents rely on the risk–benefit ratio. Parents who believe that the perceived risk of their children contracting an infectious disease is lower than the perceived risk of vaccine efficacy are more likely to refuse vaccinations. It has also been observed that the adverse effects following vaccination among children who received the Pfizer-BioNTech mRNA COVID-19 vaccine were reportedly mild, mainly presenting as local reactions such as pain, swelling, and redness at the injection site (22%); low-grade fever (15.6%); high-grade fever (1.8%); and rhinorrhea (1%) [29]. Therefore, government organizations must effectively inform, educate, and communicate with parents to reduce the impact of negative information from varied online sources [30,31,32]. Other reasons for the administrative issues reported by one-tenth of the individuals were inaccessibility, unaffordability of the vaccine, the unavailability of the preferred vaccine, or difficulty in reaching the vaccination center. Although it was not directly related to vaccination hesitance, the shortage of vaccines and uncertainty may still cause vaccine uptake to be delayed [33]. A qualitative study reported that 8.7% of those surveyed expressed a lack of faith in the government system and said they would purchase the vaccination once it was offered on the open market. Concerns about the supply and delivery of the vaccine are related to mistrust of the government, according to other studies [21,34,35].

Previous literature has also emphasized the fact that poor knowledge regarding the vaccine, its adverse effects, efficacy, etc., increases the chances of perceiving risks and thus undermines the acceptance of the vaccine [17]. In this context, the source of information plays a vast role. Apart from genuine sources of information, social media are flooded with inauthentic information about the vaccine. The younger generation is particularly exposed to false information [36]. In the current study, the sources of information about the vaccine were mostly healthcare workers or the electronic media, as reported by most of the participants. This finding was corroborated by a study conducted in Nigeria, which regarded healthcare professionals to be the most prevalent and significant information source in a study conducted in Nigeria and the United States [24,37]. A study that aimed to know the willingness of residents of four Arabic-speaking countries, namely, Jordan, Saudi Arabia, Lebanon, and Iraq, found that the major source of information on COVID-19 vaccination was social media, which was in contrast with our study’s finding, where healthcare workers were the major source of information [38]. A qualitative study comprising key informant interviews concluded that many people are persuaded by false information about the negative consequences of the vaccine that is shared on social media [26]. Thus, empowering the general population with an accurate and accessible source of information is also a crucial step in improving acceptance. The distrust about the vaccine’s efficacy and its potential to cause adverse effects was recognized as an important reason for low acceptance in various studies [28]. The importance of raising awareness about the vaccine was demonstrated numerically through the fractional order model by Baba et al. It was emphasized that a negative attitude towards vaccination increased the proliferation of infectious diseases [39,40].

Similarly, a study conducted in Chile reported that a lack of confidence in a vaccine, negative experiences, and inconvenience were associated with refusing the vaccination [17]. Thus, the vaccine delivery system should be strengthened by increasing the trained workforce, encouraging the production and distribution of the vaccine, and ensuring the efficacy of the vaccine. One of the limitations of this study was that it was a cross-sectional study and the community’s responses were recorded at one particular study period. The population’s intention to receiving the COVID-19 vaccine can vary over time and in the context of the study population.

## 5. Conclusions

It can be concluded that vaccine hesitancy due to fear of adverse effects and long-term complications was common in the elderly, males, the population of the lower middle class, and smokers. Administrative issues were mostly cited by the elderly and individuals from the upper middle social class as barriers to vaccine hesitancy. Mistrust was common in the upper middle social class. The most common barriers identified were a fear of adverse effects and long-term complications, overcrowding, loss of daily wages, and the inability to book slots. Overall, males and smokers were identified as independent predictors of vaccine refusal. Thus, in the present study, we could identify the barriers to vaccine acceptance. This could guide stakeholders to identify the pockets of vaccine refusal and thus take proper action. This necessitates the use of succinct and precise communication to dispel misconceptions about the vaccines and its efficacy. Additionally, the availability of vaccines and vaccination sites should also be communicated to tackle the administrative issues and ease its utilization, especially among elderly.

Local bodies such as self-help groups should be mobilized to inculcate confidence about the vaccine in the general population.

## Figures and Tables

**Table 1:** Distribution of variables in the vaccinated and unvaccinated groups.

Variables	Categories	Group		Total	Chi-Square, *p*-Value
		Unvaccinated	Partially Vaccinated		
Age (in years)	<20	149 (8.9%)	212 (5.7%)	361 (6.7%)	69.583, <0.001
	21–30	817 (49.0%)	1516 (40.8%)	2333 (43.4%)	
	31–40	439 (26.3%)	1265 (34.1%)	1704 (31.7%)	
	41–50	168 (10.1%)	510 (13.7%)	678 (12.6%)	
	51–60	64 (3.8%)	141 (3.8%)	205 (3.8%)	
	>60	32 (1.9%)	68 (1.8%)	100 (1.9%)	
Gender	Female	546 (32.7%)	1531 (41.2%)	2077 (38.6%)	35.350, <0.001
	Male	1123 (67.3%)	2181 (58.8%)	3304 (61.4%)	
Place of residence	Rural	40 (2.4%)	102 (2.7%)	142 (2.6%)	0.553, 0.457
	Urban	1629 (97.6%)	3610 (97.3%)	5239 (97.4%)	
Literacy	Illiterate	502 (30.1%)	829 (22.3%)	1331 (24.7%)	96.832, <0.001
	Primary school	104 (6.2%)	162 (4.4%)	266 (4.9%)	
	Middle school	178 (10.7%)	283 (7.6%)	461 (8.6%)	
	Higher school	328 (19.7%)	831 (22.4%)	1159 (21.5%)	
	Intermediate/diploma	350 (21.0%)	879 (23.7%)	1229 (22.8%)	
	Graduate	177 (10.6%)	557 (15.0%)	734 (13.6%)	
	Postgraduate	30 (1.8%)	171 (4.6%)	201 (3.7%)	
Occupation	Unemployed	422 (25.3%)	1053 (28.4%)	1475 (27.4%)	75.397, <0.001
	Unskilled worker	318 (19.1%)	673 (18.1%)	991 (18.4%)	
	Semi-skilled worker	382 (22.9%)	568 (15.3%)	950 (17.7%)	
	Skilled worker	326 (19.5%)	672 (18.1%)	998 (18.5%)	
	Arithmetically skilled jobs	161 (9.6%)	504 (13.6%)	665 (12.4%)	
	Semi-professional	37 (2.2%)	142 (3.8%)	179 (3.3%)	
	Professional	23 (1.4%)	100 (2.7%)	123 (2.3%)	
Family income per month	<2640	226 (13.5%)	588 (15.8%)	814 (15.1%)	45.046, <0.001
	2641–7886	203 (12.2%)	508 (13.7%)	711 (13.2%)	
	7887–13,160	428 (25.6%)	665 (17.9%)	1093 (20.3%)	
	13,161–19,758	359 (21.5%)	823 (22.2%)	1182 (22.0%)	
	19,759–26,354	305 (18.3%)	759 (20.4%)	1064 (19.8%)	
	26,355–52,733	129 (7.7%)	310 (8.4%)	439 (8.2%)	
	>52,734	19 (1.1%)	59 (1.6%)	78 (1.4%)	
Modified Kuppuswamy classification	Lower	88 (5.3%)	166 (4.5%)	254 (4.7%)	29.417, <0.001
	Upper lower	880 (52.7%)	1727 (46.5%)	2607 (48.4%)	
	Lower middle	537 (32.2%)	1321 (35.6%)	1858 (34.5%)	
	Upper middle	150 (9.0%)	429 (11.6%)	579 (10.8%)	
	Upper	14 (0.8%)	69 (1.9%)	83 (1.5%)	
H/O previous COVID-19 infection?	Yes	143 (8.6%)	468 (12.6%)	611 (11.4%)	18.667, <0.001
	No	1526 (91.4%)	3244 (87.4%)	4770 (88.6%)	
Source of information on vaccines	Electronic media	633 (37.9%)	1113 (30.0%)	1746 (32.4%)	52.440, <0.001
	Print media	5 (0.3%)	23 (0.6%)	28 (0.5%)	
	Healthcare worker	607 (36.4%)	1697 (45.7%)	2304 (42.8%)	
	Family and friends	311 (18.6%)	674 (18.2%)	985 (18.3%)	
	Colleagues, peer group	105 (6.3%)	196 (5.3%)	301 (5.6%)	
	Knowledge due to medical profession	8 (0.5%)	9 (0.2%)	17 (0.3%)	
H/O smoking?	Yes	252 (15.1%)	427 (11.5%)	679 (12.6%)	13.500, <0.001
	No	1417 (84.9%)	3285 (88.5%)	4702 (87.4%)	
H/O alcohol intake?	Yes	561 (33.6%)	954 (25.7%)	1515 (28.2%)	35.635, <0.001
	No	1108 (66.4%)	2758 (74.3%)	3866 (71.8%)	
H/O comorbidities	Yes	108 (6.5%)	305 (8.2%)	413 (7.7%)	4.956, 0.026
	No	1561 (93.5%)	3407 (91.8%)	4968 (92.3%)	
Have you taken annual influenza vaccine?	Yes	19 (1.1%)	52 (1.4%)	71 (1.3%)	18.676, <0.001
	No	948 (56.8%)	2327 (62.7%)	3275 (60.9%)	
	Not aware	702 (42.1%)	1333 (35.9%)	2035 (37.8%)	

**Table 2:** Association between sociodemographic characteristics and reasons for vaccine hesitancy related to fear and mistrust among unvaccinated individuals.

Variables	Categories	Fear		Chi-Square, *p*-Value	Mistrust		Chi-Square, *p*-Value
		Yes	No		Yes	No	
Age (in years)	<20	140 (94.0%)	9 (6.0%)	19.811, 0.001	112 (75.2%)	37 (24.8%)	22.698, <0.001
	21–30	726 (88.9%)	91 (11.1%)		491 (60.1%)	326 (39.9%)	
	31–40	407 (92.7%)	32 (7.3%)		274 (62.4%)	165 (37.6%)	
	41–50	159 (94.6%)	9 (5.4%)		100 (59.5%)	68 (40.5%)	
	51–60	57 (89.1%)	7 (10.9%)		29 (45.3%)	35 (54.7%)	
	>60	24 (75.0%)	8 (25.0%)		15 (46.9%)	17 (53.1%)	
Gender	Female	483 (88.5%)	63 (11.5%)	4.600, 0.032	330 (60.4%)	216 (39.6%)	0.184, 0.668
	Male	1030 (91.7%)	93 (8.3%)		691 (61.5%)	432 (38.5%)	
Place of residence	Rural	32 (80.0%)	8 (20.0%)	5.489, 0.019	27 (67.5%)	13 (32.5%)	0.690, 0.406
	Urban	1481 (90.9%)	148 (9.1%)		994 (61.0%)	635 (39.0%)	
Literacy	Illiterate	469 (93.4%)	33 (6.6%)	23.897, 0.001	306 (61.0%)	196 (39.0%)	6.181, 0.403
	Primary school	97 (93.3%)	7 (6.7%)		64 (61.5%)	40 (38.5%)	
	Middle school	160 (89.9%)	18 (10.1%)		108 (60.7%)	70 (39.3%)	
	Higher school	292 (89.0%)	36 (11.0%)		202 (61.6%)	126 (38.4%)	
	Intermediate/diploma	324 (92.6%)	26 (7.4%)		228 (65.1%)	122 (34.9%)	
	Graduate	146 (82.5%)	31 (17.5%)		97 (54.8%)	80 (45.2%)	
	Postgraduate	25 (83.3%)	5 (16.7%)		16 (53.3%)	14 (46.7%)	
Occupation	Unemployed	376 (89.1%)	46 (10.9%)	26.113, <0.001	252 (59.7%)	170 (40.3%)	49.160, < 0.001
	Unskilled worker	271 (85.2%)	47 (14.8%)		146 (45.9%)	172 (54.1%)	
	Semi-skilled worker	348 (91.1%)	34 (8.9%)		242 (63.4%)	140 (36.6%)	
	Skilled worker	311 (95.4%)	15 (4.6%)		226 (69.3%)	100 (30.7%)	
	Arithmetically skilled jobs	153 (95.0%)	8 (5.0%)		113 (70.2%)	48 (29.8%)	
	Semi-professional	32 (86.5%)	5 (13.5%)		25 (67.6%)	12 (32.4%)	
	Professional	22 (95.7%)	1 (4.3%)		17 (73.9%)	6 (26.1%)	
Family income per month	<2640	215 (95.1%)	11 (4.9%)	142.783, <0.001	219 (96.9%)	7 (3.1%)	576.273, <0.001
	2641–7886	195 (96.1%)	8 (3.9%)		191 (94.1%)	12 (5.9%)	
	7887–13,160	410 (95.8%)	18 (4.2%)		319 (74.5%)	109 (25.5%)	
	13,161–19,758	342 (95.3%)	17 (4.7%)		202 (56.3%)	157 (43.7%)	
	19,759–26,354	226 (74.1%)	79 (25.9%)		56 (18.4%)	249 (81.6%)	
	26,355–52,733	107 (82.9%)	22 (17.1%)		29 (22.5%)	100 (77.5%)	
	>52,734	18 (94.7%)	1 (5.3%)		5 (26.3%)	14 (73.7%)	
Modified Kuppuswamy classification	Lower	86 (97.7%)	2 (2.3%)	29.608, <0.001	85 (96.6%)	3 (3.4%)	122.792, <0.001
	Upper lower	822 (93.4%)	58 (6.6%)		598 (68.0%)	282 (32.0%)	
	Lower middle	464 (86.4%)	73 (13.6%)		274 (51.0%)	263 (49.0%)	
	Upper middle	128 (85.3%)	22 (14.7%)		56 (37.3%)	94 (62.7%)	
	Upper	13 (92.9%)	1 (7.1%)		8 (57.1%)	6 (42.9%)	
H/O previous COVID-19 infection?	Yes	129 (90.2%)	14 (9.8%)	0.036, 0.849	104 (72.7%)	39 (27.3%)	8.789, 0.003
	No	1384 (90.7%)	142 (9.3%)		917 (60.1%)	609 (39.9%)	
Source of information on vaccines	Electronic media	599 (94.6%)	34 (5.4%)	35.497, <0.001	316 (49.9%)	317 (50.1%)	104.776, <0.001
	Print media	4 (80.0%)	1 (20.0%)		4 (80.0%)	1 (20.0%)	
	Healthcare worker	529 (87.1%)	78 (12.9%)		370 (61.0%)	237 (39.0%)	
	Family and friends	270 (86.8%)	41 (13.2%)		224 (72.0%)	87 (28.0%)	
	Colleagues, peer group	104 (99.0%)	1 (1.0%)		101 (96.2%)	4 (3.8%)	
	Knowledge due to medical profession	7 (87.5%)	1 (12.5%)		6 (75.0%)	2 (25.0%)	
H/O smoking?	Yes	237 (94.0%)	15 (6.0%)	4.036, 0.045	130 (51.6%)	122 (48.4%)	11.486, 0.001
	No	1276 (90.0%)	141 (10.0%)		891 (62.9%)	526 (37.1%)	
H/O alcohol intake?	Yes	496 (88.4%)	65 (11.6%)	5.002, 0.025	313 (55.8%)	248 (44.2%)	10.302, 0.001
	No	1017 (91.8%)	91 (8.2%)		708 (63.9%)	400 (36.1%)	
H/O comorbidities	Yes	97 (89.8%)	11 (10.2%)	0.096, 0.757	67 (62.0%)	41 (38.0%)	0.036, 0.849
	No	1416 (90.7%)	145 (9.3%)		954 (61.1%)	607 (38.9%)	

**Table 3:** Logistic regression to predict factors associated with vaccine hesitancy.

Variables	Category	Univariate		Multivariate	
		*p*-Value	OR (95% CI)	*p*-Value	OR (95% CI)
Age (in years)	<20 (Ref)	-	-	-	-
	21–30	<0.001	3.11 (1.72–5.61)	0.015	2.15 (1.16–3.97)
	31–40	0.001	2.89 (1.57–5.33)	0.070	1.81 (0.95–3.44)
	41–50	0.004	2.75 (1.39–5.43)	0.061	1.99 (0.97–4.10)
	51–60	<0.001	4.09 (1.86–9.00)	0.012	3.08 (1.28–7.39)
	>60	0.001	4.76 (1.86–12.17)	0.002	6.52 (1.98–21.46)
Gender	Male (Ref)	-	-		
	Female	0.167	1.19 (0.93–1.52)		
Place of residence	Rural (Ref)	-	-		
	Urban	0.580	0.81 (0.39–1.68)		
Literacy	Illiterate (Ref)	-	-	0.000	
	Primary/middle/high school/diploma	0.134	0.82 (0.62–1.07)	0.061	0.71 (0.50–1.02)
	Graduate/above	<0.001	1.92 (1.35–2.75)	0.026	1.86 (1.08–3.22)
Occupation	Semi-professional/professional (Ref)	-	-		
	Unemployed	0.111	1.83 (0.87–3.84)		
	Unskilled/semi-skilled/skilled/arithmetically skilled	0.286	1.48 (0.72–3.05)		
Income	≤7886 (Ref)	-	-	0.000	
	7887–26,354	<0.001	15.71 (8.00–30.83)	<0.001	16.76 (8.40–33.43)
	>26,355	<0.001	46.67 (22.38–97.29)	<0.001	88.23 (35.83–217.28)
SES	Lower/upper lower (Ref)	-	-	0.000	
	Lower middle	<0.001	2.21 (1.71–2.85)	0.059	1.39 (0.99–1.97)
	Upper middle/upper	<0.001	2.77 (1.92–4.01)	0.003	0.33 (0.16–0.68)
H/O Previous COVID-19 infection	No (Ref)	-	-		
	Yes	0.157	0.72 (0.46–1.13)		
H/O smoking	No (Ref)	-	-		
	Yes	<0.001	2.18 (1.63–2.92)	<0.001	2.20 (1.59–3.04)
H/O alcohol intake	No (Ref)	-	-		
	Yes	<0.001	1.65 (1.30–2.10)		
H/O comorbidities	No (Ref)	-	-		
	Yes	0.353	1.24 (0.79–1.95)		

## Data Availability

The data will be available with corresponding author and will made available on request via email.
